# Spiritual well-being of terminally ill patients and next-of-kin caregivers in hospice care: A quantitative and qualitative approach

**DOI:** 10.1017/S1478951525000409

**Published:** 2025-04-22

**Authors:** Er-Jung Hsueh, Shu-Chun Tsai, Jun-Hung Lai, Chi-Yu Lu, Tsai-Wei Huang, Made Satya Nugraha Gautama

**Affiliations:** 1 Cancer Center, Yuan’s General Hospital, Kaohsiung, Taiwan; 2 Hemophilia Center, Yuan’s General Hospital, Kaohsiung, Taiwan; 3 Division of Hemato-Oncology, Department of Internal Medicine, Yuan’s General Hospital, Kaohsiung, Taiwan; 4 School of Nursing, College of Nursing, Taipei Medical University, Taipei City, Taiwan; 5 Department of Nursing, Wan Fang Hospital, Taipei Medical University, Taipei City, Taiwan; 6 Division of Gastroenterology & Hepatology, Department of Internal Medicine, Erlin Christian Hospital, Changhua, Taiwan; 7 Research Center in Nursing Clinical Practice, Wan Fang Hospital, Taipei Medical University, Taipei City, Taiwan; 8 Cochrane Taiwan, Taipei Medical University, Taipei City, Taiwan; 9 Department of Nursing, Faculty of Medicine, Universitas Pendidikan Ganesha, Singaraja, Bali, Indonesia

**Keywords:** Terminal cancer patients, next-of-kin caregivers, spiritual well-being, hospice palliative care, gender differences, holistic care

## Abstract

**Background:**

Terminal cancer patients often endure significant distress, impacting their quality of life. Spiritual well-being provides peace and meaning during this challenging period.

**Objectives.** This study explored the spiritual well-being of terminally ill patients and their next-of-kin caregivers in hospice care, focusing on factors influencing their spiritual experiences.

**Methods:**

This mixed-methods study included 30 terminally ill patients and 17 next-of-kin caregivers in hospice care. Spiritual well-being was assessed using the Functional Assessment of Chronic Illness Therapy – Spiritual Well-Being Scale (FACIT-Sp-12), and symptom distress with the Edmonton Symptom Assessment Scale. Qualitative data were collected through semi-structured interviews at baseline, 1 week, and 1 month. Data were analyzed using quantitative methods and thematic analysis.

**Results:**

Patients showed a significant improvement in spiritual well-being over time, with FACIT-Sp-12 scores increasing from 28.6 at baseline to 31.3 at 1 month (*p* < .01). Symptoms such as shortness of breath (*β* = –1.19, *p* < .001), drowsiness (*β* = –1.27, *p* = .01), and anxiety (*β* = –0.60, *p* = .03) were negatively associated with spiritual well-being. Caregiver spiritual well-being positively influenced patient scores, especially with female caregivers (*β* = 0.26, *p* < .001). Qualitative findings supported these results, revealing themes of spiritual adjustment, the impact of physical symptoms on spiritual well-being, and the crucial role of caregivers in providing emotional and spiritual support.

**Significance of results:**

Early palliative care facilitates spiritual adjustment in terminally ill patients. A holistic approach addressing physical symptoms and psychological distress is essential. Supporting caregivers, particularly female ones, positively impacts patient spiritual well-being. Tailored interventions considering the unique needs of patients and caregivers are recommended to enhance palliative care quality.

## Graphical abstract

**Figure:**
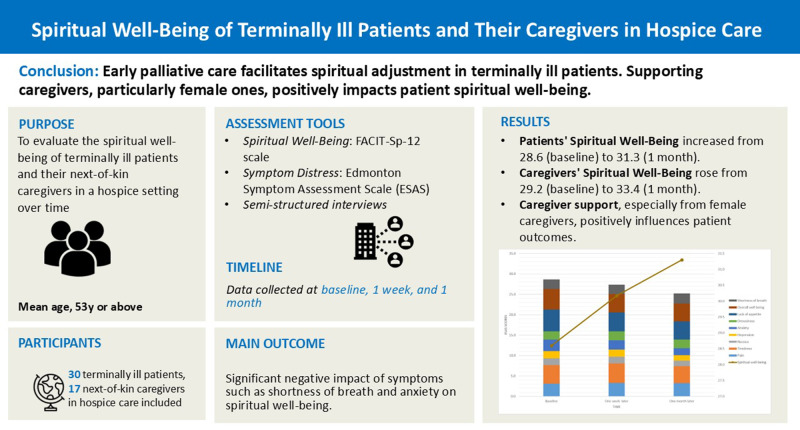

## Introduction

Terminal cancer profoundly affects patients’ quality of life (QoL), inducing not only physical symptoms but also significant psychological and existential distress. While medical advances have made it possible to manage many physical symptoms associated with terminal cancer, emotional and spiritual distress often remains inadequately addressed (Hugar et al. 2021; Meier 2011). The psychological state of patients plays a crucial role in shaping their perceptions of life and death, and neglecting this aspect can lead to increased feelings of isolation and unresolved existential distress (Razban et al. 2022). Spiritual well-being, which encompasses the search for meaning, purpose, and connection in life, is a vital component of holistic health, particularly in the context of terminal illness. It significantly influences patients’ experiences during their final stages, providing a source of comfort and peace (Ryff 2021). The World Health Organization has recognized spiritual well-being as an essential aspect of health (Dezutter et al. 2017; World Health Organization 2024 ). However, spiritual distress, marked by a sense of disharmony and existential pain, often intensifies during periods of severe stress and uncertainty, particularly for those facing terminal illness (Hayden et al. 2022). For cancer patients, spiritual well-being is critical throughout the disease trajectory, often serving as a key factor in their overall well-being and QoL (Smith 2017). Spirituality can be understood through various dimensions such as meaning, peace, and faith, each reflecting different aspects of an individual’s spiritual experience (Jimenez-Fonseca et al. 2018). These dimensions may vary in their importance depending on the stage of the disease, necessitating a personalized approach to spiritual care.

In Eastern cultures, including Taiwan, familial bonds play a significant role in the experience of illness and care. Caregivers, often next-of-kin family members, are deeply involved in the patient’s journey, and their own spiritual well-being can influence that of the patient. Research has highlighted the importance of caregiver involvement and attachment in supporting patient well-being, yet the complex dynamics of shared spirituality between patients and their next-of-kin caregivers remain underexplored (Ozdemir et al. 2024). This gap in understanding the spiritual interdependence within the patient–caregiver dyad is particularly critical in hospice settings, where spiritual well-being can profoundly influence both the patient’s and caregiver’s experience of the end of life. Therefore, there is a need to explore the spiritual experiences of terminally ill patients and their next-of-kin caregivers in hospice care. By examining both quantitative measures and qualitative feedback over time, this study aims to provide a deeper understanding of the factors influencing spiritual well-being. Specifically, the study seeks to identify how physical and psychological symptoms, caregiver spiritual well-being, and gender differences impact the spiritual well-being of terminally ill patients. This research will contribute to the development of holistic end-of-life care strategies that address the interconnected needs of patients and their caregivers, ultimately enhancing the QoL during the final stages of life.

## Methods

### Design

This study employed a parallel mixed-methods design, integrating quantitative assessments with qualitative feedback from both terminally ill patients and their next-of-kin caregivers. Spiritual well-being was assessed at baseline, and longitudinal monitoring was conducted at 1-week and 1-month follow-ups.

### Setting and participants

The study protocol was approved by the Joint Institute Research Board of Taipei Medical University, Taiwan (Institutional Review Board file no.: N201807005). Participants were recruited from an affiliated university hospital between August 2018 and August 2020. Dyads comprising terminally ill patients and their primary next-of-kin caregivers were invited to participate in this study. Written informed consent was obtained from all participants prior to study commencement.

The inclusion criteria for patients were as follows: (1) Ability to provide informed consent, no hearing problems, and ability to communicate in Mandarin or Taiwanese; (2) age ≥20 years; (3) terminal diagnosis with a life expectancy of ≤6 months, and (4) willingness to be interviewed and recorded. The corresponding criteria for next-of-kin caregivers were as follows: (1) Ability to provide informed consent, no hearing problems, and ability to communicate in Mandarin or Taiwanese; (2) age ≥20 years. This threshold was set considering legal adulthood in Taiwan and the ability to independently provide caregiving decisions; (3) primary caregiving role for the patient; and (4) willingness to be interviewed and recorded.

### Spiritual support services in hospice care

Spiritual care in the hospice unit was delivered by a multidisciplinary team, including professional chaplains representing diverse religious traditions, psycho-oncology specialists, and nurses and volunteers trained in spiritual care. Patients and caregivers had access to the following services: (1) Weekly individual chaplain visits based on patient needs, with an average of 1–2 visits during hospitalization; (2) weekly group spiritual support meetings facilitated by the hospice team; (3) a dedicated quiet space within the unit for prayer and reflection; and (4) relaxation technique assistance for families upon request. As part of the study protocol, all participants received at least one chaplain visit during the study period and participated in at least one group spiritual support meeting. All spiritual care services respected Taiwan’s diverse religious context, with a particular focus on Buddhist and Taoist traditions, which were predominant in our patient population. Religious beliefs were assessed by directly asking participants, “Do you have religious beliefs? If yes, which religion?” Response options included “No religious belief,” “Buddhism,” “Taoism,” “Christianity,” “Catholicism,” and “Other.” These categories reflect the predominant religious traditions in Taiwan.

### Data collection

Data collection involved both quantitative and qualitative measures.

#### Quantitative assessments

Patients and their primary next-of-kin caregivers completed the Functional Assessment of Chronic Illness Therapy – Spiritual Well-Being Scale (FACIT-Sp-12) and participated in face-to-face interviews, which included open-ended questions, conducted in the oncology hospice care unit. Concurrently, patients’ symptoms were assessed using the Edmonton Symptom Assessment Scale (ESAS). Demographic information was collected through direct inquiry and review of medical records. These assessments were conducted at baseline and followed up at 1-week and 1-month intervals.

The FACIT-Sp-12 is a validated measure of spiritual well-being, particularly within the Taiwanese population (Wang and Lin 2016). It consists of 12 items divided into 2 subscales: Faith and Meaning/Peace, and it includes an open-ended question to assess overall spirituality (Brady et al. 1999). The Taiwanese version has demonstrated robust reliability with a Cronbach’s *α* of 0.84 (Wang and Lin 2016). The Faith subscale (4 items) measures spiritual comfort and strength derived from faith, while the Meaning/Peace subscale (8 items) evaluates perceptions of meaning, peace, and purpose in life. Responses are rated on a 5-point Likert scale ranging from 0 (*not at all*) to 4 (*very much*), with total scores ranging from 0 to 48. Higher scores indicate greater spiritual well-being.

The ESAS is a widely used tool that evaluates 9 core symptoms: Pain, fatigue, drowsiness, lack of appetite, shortness of breath (SOB), nausea, anxiety, depression, and well-being (Chow et al. 2019). Each symptom is rated on a scale from 0 to 10, with 0 indicating no occurrence and 10 indicating the most severe condition. The ESAS effectively monitors both acute and chronic symptom burdens, providing valuable insights into patients’ symptom trajectories over time (Hamer et al. 2017).

#### Qualitative interview

At each assessment point, patients and caregivers participated in semi-structured interviews to explore their subjective experiences of spiritual well-being and symptom distress. Open-ended questions were designed to elicit their perspectives on the meaning of their spiritual well-being scores and factors influencing their spiritual peace. Interview prompts included questions like, “How would you describe your current spiritual well-being?” and “What factors contribute to or hinder your sense of peace?” (Steinhauser et al. 2006). When participants had difficulty articulating their spiritual state, a 0–10 scale was provided to facilitate more precise responses, allowing for culturally appropriate, indirect expressions of spiritual distress or contentment common in Taiwanese communication norms.

### *Statistical analy*sis

#### Quantitative analysis

Descriptive statistics were calculated for demographic characteristics, FACIT-Sp-12, and ESAS scores. Generalized estimating equation (GEE) analyses were conducted to account for the correlation in repeated measurements over time. All analyses were performed using IBM SPSS Statistics for Windows, Version 22.0 (IBM Corp., Armonk, NY, USA), with significance set at *p* < .05 (2-sided).

#### Qualitative analysis

Thematic analysis was applied to qualitative data from interviews. All responses were transcribed and coded to identify common themes and differences. Key themes focused on the subjective meanings of spiritual well-being, the impact of symptoms on peace, and the influence of caregiver presence. This approach provided depth to the quantitative findings, offering insight into the personal and relational factors affecting spiritual well-being at end-of-life.

## Results

**Figure 1.**
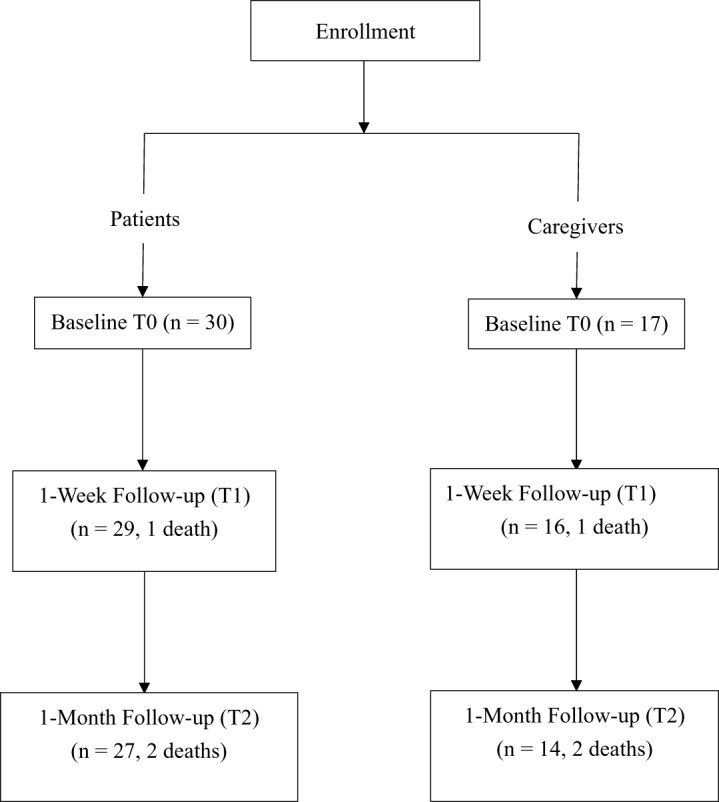
Participant recruitment flowchart.

A total of 30 patients and 17 next-of-kin caregivers were initially enrolled in the study (Fig. 1). Over the course of the study, one patient passed away within the first week, followed by the deaths of 2 additional patients after the first week. Consequently, 29 patients and 16 caregivers were available for assessment at the 1-week follow-up. By the 1-month follow-up, the number of participants had further decreased to 27 patients and 14 caregivers. The demographic and clinical characteristics of the study participants are summarized in Table 1. The patient cohort was predominantly female (76.7%), with a mean age of 62.9 years. The majority of patients were married (60%), and their education levels ranged from senior high school to a master’s degree. A significant portion of patients (73.3%) reported having religious beliefs. The most prevalent cancer types in this group were breast cancer (26.7%), colorectal cancer (16.7%), and ovarian/cervical cancer (13.3%). Other cancers included lung, hepatic/pancreatic, gastric, head and neck, osteosarcoma, bladder, prostate, and multiple myeloma.

**Table 1.** Demographic and clinical characteristics of patients and caregivers (*n* = 30)

	Total (*n* = 30)		Patients with company (*n* = 17)		Patients without company (*n* = 13)		Next-of-kin caregivers (*n* = 17)	
Characteristic	Mean	SD	Mean	SD	Mean	SD	Mean	SD
Age	62.9	12.98	66.3	10.70	58.5	14.72	50.0	17.51
	*n*	%	*n*	%	*n*	%	*n*	%
Sex								
Male	7	23.3	4	23.5	3	23.1	3	17.6
Female	23	76.7	13	76.5	10	76.9	14	82.4
Marital status								
Married	18	60.0	12	70.6	6	46.2	6	35.3
Unmarried	12	40.0	5	29.4	7	53.8	11	64.7
Education								
Senior high school or under	16	53.3	13	76.5	3	23.0	4	23.5
College	10	33.3	4	23.5	6	46.2	11	64.7
Master’s degree	4	13.3	0	0	4	30.8	2	11.8
Religious belief								
Buddhism	9	30	5	29.4	4	30.8	5	29.4
Taoism	8	26.7	6	35.3	2	15.4	4	23.5
Christianity	4	13.3	1	5.9	3	23.1	3	17.6
Catholicism	1	3.3	1	5.9	0	30.8	1	5.9
No	8	26.7	4	23.5	4	30.8	4	23.5
Primary cancer type								
Breast	8	26.7	3	17.6	5	38.5		
Colorectal	5	16.7	5	29.4	0	0		
Ovarian/cervical	4	13.3	1	5.9	3	23.1		
Lung	3	10.0	2	11.8	1	7.7		
Hepatic/pancreatic	3	10.0	2	11.8	1	7.7		
Gastric	2	6.7	2	11.8	0	0		
Head and neck	1	3.3	0	0	1	7.7		
Osteosarcoma	1	3.3	1	5.9	0	0		
Bladder	1	3.3	1	5.9	0	0		
Prostate	1	3.3	0	0	1	7.7		
Multiple myeloma	1	3.3	0	0	1	7.7		
Relationship								
Spouse							4	23.5
Son or daughter							7	41.2
Parents							2	11.8
Others							4	23.5

The next-of-kin caregivers had a mean age of 50.0 years and had been providing care for an average of 3.7 years. Most caregivers were female (82.4%) and reported having religious beliefs (76.5%). Their relationships to the patients varied, with the majority being sons or daughters (41.2%), followed by spouses (23.5%).

Over time, patients exhibited a gradual improvement in spiritual well-being, as reflected by increases in FACIT-Sp-12 scores. The total spiritual well-being score rose from 28.6 (SD = 8.05) at baseline to 31.3 (SD = 8.79) at the 1-month follow-up, indicating an overall enhancement in their spiritual state. Both the Meaning/Peace and Faith subscales showed similar improvements, supporting the notion that spiritual care positively impacts patients in palliative care (Table 2).

**Table 2.** Symptom distress and spiritual well-being among patients and caregivers

Assessment	Baseline	1 week	1 month
Patients	*n* = 30, mean (SD)	*n* = 29, mean (SD)	*n* = 27, mean (SD)
FACIT-Sp			
Meaning/peace	2.2 (0.63)	2.3 (0.64)	2.4 (0.79)
Faith	2.7 (0.87)	2.9 (0.87)	2.9 (0.83)
Total score	28.6 (8.05)	30.2 (8.11)	31.3 (8.79)
ESAS			
Pain	3.1 (3.16)	3.3 (2.84)	3.2 (2.64)
Tiredness	4.5 (3.09)	4.8 (3.03)	4.2 (2.92)
Nausea	1.7 (2.77)	1.6 (2.27)	1.3 (2.00)
Depression	1.8 (2.45)	1.8 (1.98)	1.3 (1.92)
Anxiety	2.8 (2.73)	2.3 (2.22)	1.7 (2.07)
Drowsiness	2.0 (2.89)	2.2 (2.84)	2.1 (3.02)
Lack of appetite	5.4 (3.11)	4.7 (3.33)	4.5 (3.56)
Overall well-being	5.0 (3.04)	4.5 (2.75)	4.4 (3.24)
Shortness of breath	2.4 (2.89)	2.3 (2.66)	2.4 (3.18)
Next-of-kin caregivers	*n* = 17	*n* = 16	*n* = 14
FACIT-Sp			
Meaning/peace	2.4 (0.60)	2.5 (0.69)	2.8 (0.80)
Faith	2.5 (1.39)	2.5 (1.39)	2.8 (1.28)
Total score	29.2 (9.63)	30.2 (10.37)	33.4 (10.45)

ESAS = Edmonton Symptom Assessment Scale; FACIT-Sp-12 = Functional Assessment of Chronic Illness Therapy – Spiritual Well-Being Scale.

**Figure 2.**
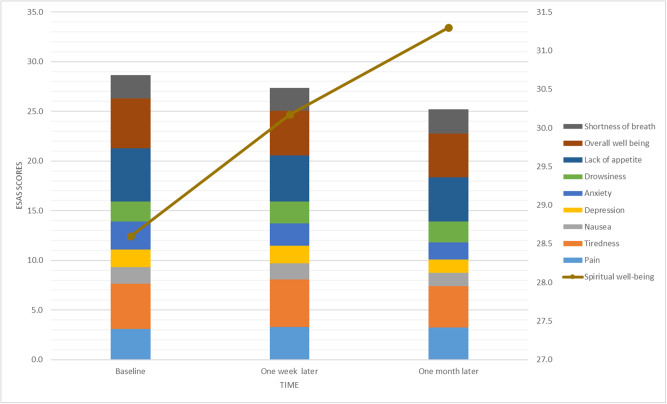
Changes in symptom distress and spiritual well-being among hospice patients over time.

In terms of symptom distress, the baseline assessments revealed that lack of appetite (mean = 5.4, SD = 3.11), overall well-being (mean = 5.0, SD = 3.04), and tiredness (mean = 4.5, SD = 3.09) were the most severe symptoms. However, these symptoms showed slight improvements over time, with lack of appetite decreasing to 4.5 (SD = 3.56) and overall well-being improving to 4.4 (SD = 3.24) by the 1-month follow-up (Fig. 2).

Next-of-kin caregivers also experienced an increase in spiritual well-being. Their FACIT-Sp-12 scores increased from 29.2 (SD = 9.63) at baseline to 33.4 (SD = 10.45) at 1 month. The improvements were consistent across both the Meaning/Peace and Faith subscales, indicating that caregivers, like patients, benefitted from the spiritual care provided during hospice.

### Qualitative analysis

The qualitative analysis revealed several themes highlighting both shared and distinct perspectives between patients and caregivers. Patients often focused on managing physical symptoms and processing their acceptance of mortality, whereas caregivers expressed concern for the patient’s condition and the psychological pressures associated with caregiving responsibilities. Key themes included:

- *Enhanced Spiritual Comfort*: Patients and caregivers reported a sense of peace after engaging in spiritual care. For instance, a caregiver stated, “Receiving psychological support helped me feel more at ease” (Caregiver of P2).
- *Symptom Distress*: Patients commonly mentioned that pain and discomfort negatively impacted their spiritual well-being. One patient shared, “The pain affects my appetite and mood” (Patient P5).
- *Caregiver Anxiety and Support Needs*: Caregivers expressed concerns about the patient’s condition and their own challenges in coping. A caregiver mentioned, “There are so many difficulties and challenges still ahead” (Caregiver of P5).
- *Faith and Acceptance*: Some patients and caregivers found peace through faith. A patient noted, “I have let go and left everything to a higher power” (Patient P18).
- *Fear and Uncertainty*: Both patients and caregivers conveyed anxiety about the future. A patient described their apprehension, stating, “The future is unknown, and I feel fearful” (Patient P29).

### Quantitative analysis

The GEE analysis (Table 3) revealed that male patients and those with religious beliefs had significantly higher spiritual well-being scores, increasing by 11.30 points (*p* < .001) and 7.34 points (*p* = .02), respectively. Conversely, male caregivers were associated with a reduction in patients’ spiritual well-being by 5.61 points (*p* = .01). Certain cancer types, particularly bladder (*β* = −18.58, *p* < .001), gastric (*β* = −27.38, *p* < .001), and colorectal cancers (*β* = −13.87, *p* < .001), were linked with a significant reduction in spiritual well-being.

**Table 3.** GEE results indicating factors associated with patient spiritual well-being

Characteristic	*β*	95% CI	Wald	*p*
Patient characteristics				
Male sex (ref: female sex)	11.30	6.5 to 16.1	15.43	<.001
Religious belief (ref: none)	7.34	1.3 to 13.4	5.21	.02
Caregiver characteristics				
Male sex (ref: female sex)	−5.61	−10.9 to −0.3	6.58	.01
Timeline	1.76	0.5 to 3.0	7.45	.01
Caregiver spiritual well-being	0.26	0.1 to 0.4	12.56	<.001
ESAS symptoms				
Pain	0.27	−0.2 to 0.8	1.03	.31
Tiredness	−0.36	−0.9 to 0.2	1.58	.21
Nausea	0.59	−0.2 to 1.3	2.32	.13
Depression	0.85	−0.3 to 2.0	2.22	.14
Anxiety	−0.60	−1.1 to −0.1	4.97	.03
Drowsiness	−1.27	−2.3 to −0.3	6.06	.01
Lack of appetite	0.11	−0.9 to 1.1	0.05	.82
Overall well-being	−0.25	−0.9 to 0.4	0.55	.46
Shortness of breath	−1.19	−1.8 to −0.6	16.67	<.001

ESAS = Edmonton Symptom Assessment Scale; GEE = generalized estimating equation.

Importantly, spiritual well-being scores improved over time (*β* = 1.76, *p* = .01), suggesting that ongoing palliative care plays a beneficial role in enhancing patients’ spiritual well-being. Additionally, the spiritual well-being of caregivers had a positive impact on the patients’ spiritual well-being (*β* = 0.26, *p* < .001), highlighting the interconnectedness of patient and caregiver experiences.

Among the physical and psychological symptoms, SOB (*β* = −1.19, *p* < .001), drowsiness (*β* = −1.27, *p* = .01), and anxiety (*β* = −0.60, *p* = .03) were significantly negatively associated with spiritual well-being, while other symptoms such as pain, tiredness, nausea, depression, lack of appetite, and overall well-being did not show significant effects. These findings underscore the necessity of addressing both spiritual and symptom management in palliative care to improve the overall well-being of terminally ill patients and their caregivers (Table 3 and Fig. S1).

## Discussion

This study found that patients in hospice care experienced moderate improvements in spiritual well-being over the study period. Notably, male patients and those with religious beliefs reported higher levels of spiritual well-being compared to their counterparts. Conversely, patients with terminal illnesses that severely affected eating or digestive functions – such as gastric and colorectal cancers – exhibited lower levels of spiritual well-being. Among the physical and psychological symptoms assessed, SOB and anxiety had the most significant adverse effects on spiritual well-being. A decline in these symptoms over time coincided with improvements in spiritual well-being. Additionally, the spiritual well-being of caregivers positively influenced patient outcomes, with female caregivers showing a particularly strong impact.

These findings underscore the critical role of existential distress – which encompasses physical and psychological suffering – in diminishing the QoL for those nearing the end of life (Rantanen et al. 2022; Tang et al. 2016). This distress presents complex challenges for patients, families, and end-of-life care providers (Breitbart et al. 2004; Kissane 2000). Sulmasy’s (2002) biopsychosocial–spiritual model emphasizes the importance of holistic care that addresses the physical, psychological, social, and spiritual dimensions of patients, highlighting the need for integrated approaches in palliative settings (Sulmasy 2002). Furthermore, the actor–partner interdependence model highlights the interconnectedness of patient and caregiver experiences, underscoring the necessity of synchronized support strategies that address the needs of both parties (Hanna and Semple 2024; Ozdemir et al. 2024).

The influence of gender on spiritual well-being observed in this study is noteworthy. Male patients reported higher spiritual well-being, which may be influenced by cultural and psychological factors where men receive more social support and validation, thereby enhancing their spiritual well-being (Luna et al. 2019). However, this finding is contrasted by the higher spiritual well-being reported by female patients with breast cancer compared to patients with other types of cancer, except lung cancer. This challenges the assumption that male gender inherently contributes to greater spiritual well-being. The higher spiritual well-being among female breast cancer patients may be attributed to higher survival rates associated with breast cancer, reducing the immediate threat of death and preserving their spiritual state.

Conversely, female caregivers had a positive influence on patients’ spiritual well-being, likely due to their nurturing roles and emotional expressiveness, which make them more effective in providing holistic support (Newberry et al. 2013). Qualitative data from our study support this finding. For instance, one patient’s caregiver expressed, “I hope the patient and other family members can accept the progression of the illness,” highlighting the caregiver’s role in facilitating acceptance and emotional support.

SOB was found to have a particularly negative impact on spiritual well-being, often provoking significant anxiety and fear. Qualitative findings indicated that patients experiencing SOB frequently reported a profound sense of loss of control, which heightened existential distress and diminished spiritual well-being. This aligns with previous studies linking dyspnea with depressed mood and increased anxiety (Zweers et al. 2018). Effective management of SOB, potentially through interventions such as continuous deep sedation, could mitigate its negative effects on spiritual well-being (Hiratsuka et al. 2021). Our qualitative data revealed statements like “Pain affects my appetite,” emphasizing how physical symptoms exacerbate spiritual distress.

Beyond specific symptoms like SOB, the enduring somatic threat model provides an additional framework for understanding distress at the end of life for both patients and caregivers. Originally described in cardiac events by Edmondson et al. (2018), this model conceptualizes how the persistent threat of bodily dysfunction and death can trigger ongoing physiological and psychological responses that significantly impact spiritual well-being. The continuous awareness of bodily vulnerability creates a state of chronic stress that exacerbates symptom perception and amplifies existential concerns. In our hospice care context, patients experiencing symptom exacerbation frequently described heightened spiritual distress, consistent with the enduring somatic threat perspective. As one patient described, “I worry about the disease” (Patient P22), while another patient expressed, “The future is unknown, and I feel fearful” (Patient P29). These expressions of uncertainty and vigilance regarding bodily changes contribute to a cycle of distress that can be difficult to interrupt without targeted interventions addressing both physical symptoms and their psychological interpretation. Future spiritual care approaches might benefit from incorporating this model to help patients and caregivers process the distress associated with enduring somatic threats while supporting their search for meaning and peace. Understanding the goals of care is crucial, particularly among patients with high symptom burdens and diminished spiritual well-being. Patients often prioritize symptom management over life extension, especially when caregivers also perceive higher caregiving burdens (Ozdemir et al. 2024). Our qualitative data showed that some patients expressed a desire for comfort and dignity over aggressive treatments. One patient stated, “When the time comes, I hope to pass away with dignity without intubation” (Patient P4), emphasizing the importance of addressing both physical and spiritual needs. Interestingly, despite formal hospice enrollment, approximately 25% of patients continued to express interest in or hope for treatment interventions during interviews. This finding reflects the complex psychological process of accepting terminal status and cultural factors specific to Taiwan, where direct discussions about death may be considered taboo. This underscores the importance of ongoing, culturally sensitive communication about goals of care throughout the hospice journey, rather than viewing hospice enrollment as signifying complete acceptance of comfort-focused care.

Addressing psychological symptoms, particularly anxiety, is essential for improving end-of-life experiences. Palliative care interventions focusing on psychological support and symptom management have been shown to reduce anxiety and enhance overall well-being (Ferrell et al. 2017). This reinforces the significant negative impact of anxiety on spiritual well-being observed in our study and highlights the need for integrated psychological care in palliative settings. As one caregiver noted, “Received psychological support and care,” indicating the value of emotional support services for both patients and caregivers.

This study has several limitations. The small sample size, common in palliative care research, limits the generalizability of the findings. Additionally, the study period was brief due to the terminal status of the patients, constraining the evaluation of long-term factors influencing spiritual well-being. While a cancer diagnosis was not explicitly required for eligibility, all enrolled patients had cancer diagnoses. This homogeneity in diagnosis may limit the applicability of our findings to patients with non-cancer terminal illnesses. Some patients lived alone without close relatives or friends, leading to a lack of next-of-kin data for those individuals. Moreover, the variability in interview settings, with some patients and caregivers choosing joint or separate interviews, may have influenced the responses. Participants might have moderated their expressions of spiritual distress to avoid worrying their loved ones, potentially masking their actual spiritual state.

## Conclusion

This study demonstrates that integrating quantitative and qualitative data from both terminally ill patients and their caregivers provides a comprehensive understanding of the critical role of spiritual care in enhancing spiritual well-being at the end of life. Our findings indicate that spiritual well-being can improve over time with palliative care interventions but is negatively impacted by physical symptoms like SOB and psychological distress such as anxiety. Patients with cancers affecting the digestive system experienced a greater decline in spiritual well-being.

Importantly, the spiritual well-being of caregivers, especially female caregivers, positively influenced patient outcomes, highlighting the interconnectedness between patients and their caregivers. This suggests that addressing caregivers’ psychological needs is essential, as their anxiety and stress can affect patients’ spiritual well-being.

These findings underscore the necessity of early integration of palliative care to allow sufficient time for spiritual adjustment. Future nursing practices should adopt a holistic care approach that addresses both the physical and psychological distress of patients and considers the unique psycho-social-spiritual needs based on individual and gender differences. Supporting both patients and their caregivers can significantly enhance the QoL for terminally ill patients and their families.

In summary, spiritual care is essential in palliative settings. Integrating comprehensive care strategies into standard practice promotes holistic well-being, providing peace and meaning during the final stages of life.

## Supporting information

Hsueh et al. supplementary material Hsueh et al. supplementary material

## Data Availability

The research data are stored in an institutional repository and will be shared upon request by the corresponding author.

## Supplementary material

The supplementary material for this article can be found at https://doi.org/10.1017/S1478951525000409.
