# Supplementary material for: Spiritual well-being of terminally ill patients and next-of-kin caregivers in hospice care: A quantitative and qualitative approach
Source: Palliat Support Care. 2025 Apr 22;23:e95. doi: 10.1017/S1478951525000409 (PMC13166419; doi:10.1017/S1478951525000409)
Supplement: Hsueh et al. supplementary material [file S1478951525000409sup001.docx]

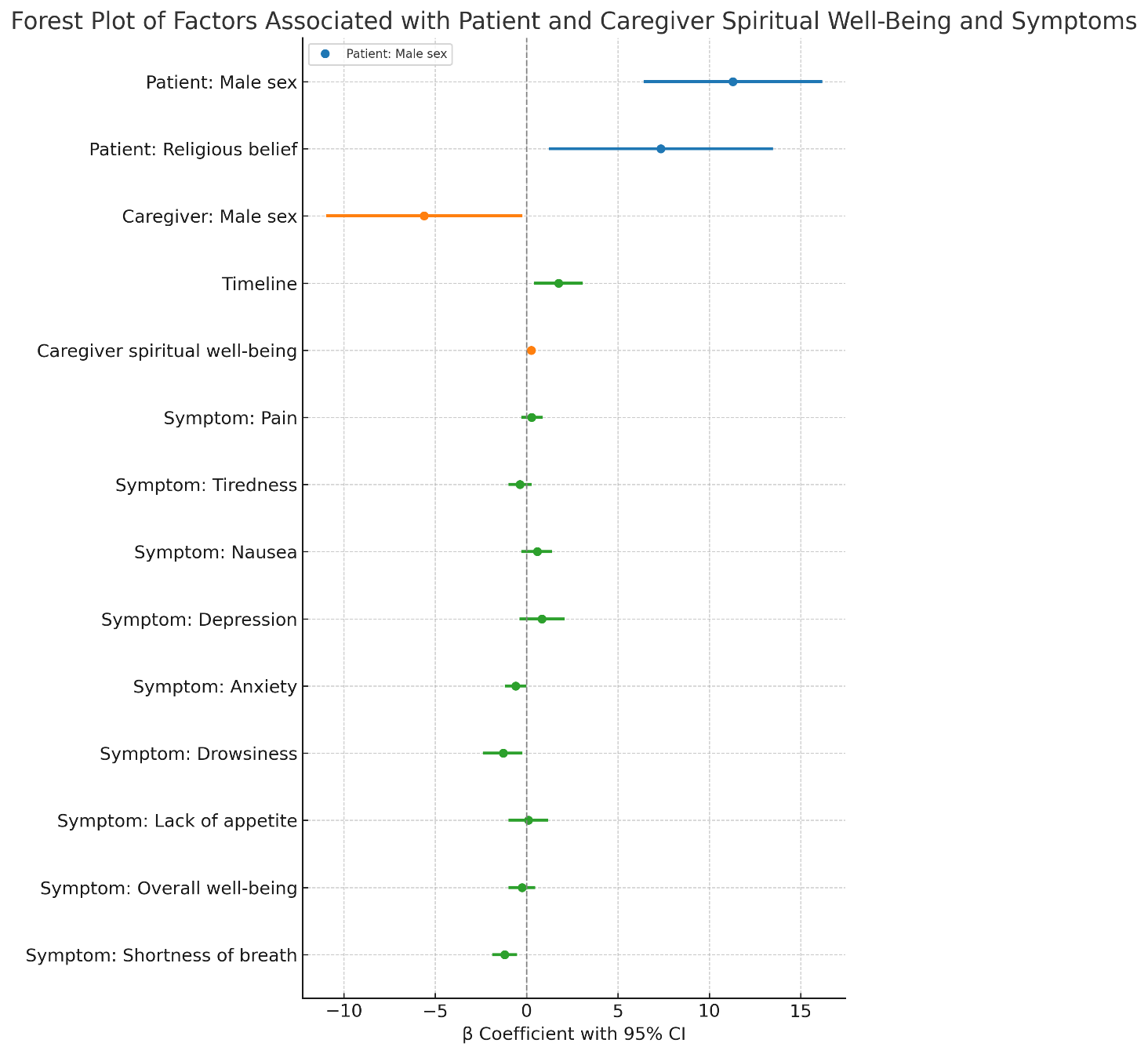


Figure S1. Forest plot of factors associated with patient and caregiver spiritual well-being and symptoms.
